# Five years follow-up following two or three doses of a hepatitis B vaccine in adolescents aged 11-15 years: a randomised controlled study

**DOI:** 10.1186/1471-2334-10-357

**Published:** 2010-12-20

**Authors:** Pierre Van Damme, Anna Moiseeva, Igor Marichev, Anne-Diane Kervyn, Robert Booy, Sherine Kuriyakose, Andrew Brockway, Su-Peing Ng, Maarten Leyssen, Jeanne-Marie Jacquet

**Affiliations:** 1 Professor, Faculty of Medicine, Vaccine & Infectious Disease Institute (WHO Collaborating Centre), Centre for the Evaluation of Vaccination, Antwerpen, Belgium; 2 Ministry of Health, Centre of Immunobiological Products, Kiev, Ukraine; 3 Centre de Santé UCL, Clos Chapelle-aux-Champs, 30/39 1200 Brussels, Belgium; 4 National Centre for Immunisation Research and Surveillance, The Children's Hospital at Westmead, NSW 2145, Australia; and The University of Sydney, NSW, Australia; 5 GlaxoSmithKline Pharmaceuticals Ltd., Bangalore, India; 6 GlaxoSmithKline Biologicals, Melbourne Victoria, Australia; 7 GlaxoSmithKline Biologicals, Singapore; 8 GlaxoSmithKline Biologicals, Wavre, Belgium

## Abstract

**Background:**

The standard three-dose schedule of hepatitis B vaccines is frequently not completed, especially in adolescents. A primary study has confirmed the equivalence of a two-dose schedule of an *Adult *formulation of hepatitis B vaccine [Group HBV_2D] to a three-dose schedule of a *Paediatric *formulation in adolescents (11-15 years) [Group HBV_3D]. This follow-up study evaluated the five year persistence of antibody response and immune memory against the hepatitis B surface (anti-HBs) antigens five years after completion of primary vaccination.

**Methods:**

A total of 234 subjects returned at the Year 5 time point, of which 144 subjects received a challenge dose of hepatitis B vaccine. Blood samples were collected yearly and pre- and post-challenge dose to assess anti-HBs antibody concentrations.

**Results:**

At the end of five years, 79.5% (95% confidence interval [CI]: 71.7 - 86.1) and 91.4% (95% CI: 82.3 - 96.8) of subjects who received the two-dose and three-dose schedules, respectively had anti-HBs antibody concentrations ≥10 mIU/mL. Post-challenge dose, all subjects had anti-HBs antibody concentration ≥10 mIU/mL and >94% subjects had anti-HBs antibody concentration ≥100 mIU/mL. All subjects mounted a rapid anamnestic response to the challenge dose. Overall, the challenge dose was well-tolerated.

**Conclusion:**

The two-dose schedule of hepatitis B vaccine confers long-term immunogenicity and shows evidence of immune memory for at least five years following vaccination.

**Trial registration:**

Clinical Trials NCT00343915, NCT00524576

## Background

Hepatitis B viral infections continue to be a serious global health problem and are a cause of concern for public health authorities [1]. The virus is estimated to have infected two billion people around the world, of whom approximately 360 million are chronically infected. These chronically infected individuals are at increased risk of developing serious illness, which may progress to liver cirrhosis and hepatocellular carcinoma (HCC), that account for an estimated annual 500,000-700,000 deaths worldwide [1]. A number of studies conducted in different countries have confirmed that universal immunisation of infants and/or adolescents is the most efficient method of reducing the disease burden of hepatitis B infection [2-5]. Taking into consideration the morbidity and mortality associated with viral hepatitis worldwide, the World Health Organization (WHO) recommended in 1992 that vaccination against hepatitis B should be included into the national immunisation schedules of all countries worldwide by 1997 [1].

The three-dose schedule of hepatitis B vaccination has been the standard immunisation schedule of choice. In countries with an adolescent hepatitis B immunisation programme, the completion rates, however, for the three-dose schedule appear to be lower than expected in certain target populations, such as adolescents [6,7]. In addition, when compared to the option of a two-dose schedule, the three-dose schedule puts a heavier burden on the healthcare system in terms of the implementation and organisation of vaccination programmes. Hence, there has been a growing interest among public healthcare authorities and vaccine manufacturers in identifying a suitable two-dose immunisation schedule that is more convenient for use in adolescents to ensure higher completion rates [7-9].

A two-dose schedule (0, 6 months) of a hepatitis B vaccine (*Engerix*-B™: *Adult *formulation, GlaxoSmithKline [GSK] Biologicals, Belgium) has been approved for use in European adolescents and is also one of the recommended schedules for vaccination of adolescents aged 11-15 years in Australia [10], United States and Canada. In addition, a three-dose schedule of the *Paediatric *formulation of this vaccine is recommended for use in children and young adults aged <20 years. A previous study in children and adolescents has demonstrated equivalence between a two-dose primary vaccination schedule of the *Adult *formulation and a three-dose schedule of the *Paediatric *formulation of this vaccine in terms of seroprotection against hepatitis B infection [8,11,12]. Considering that the risk of acquiring hepatitis B infections is higher during early adulthood due to various lifestyle-related exposure [13], it is critical to assess the long-term persistence of vaccine-induced immunity in young adults who have been vaccinated with hepatitis B vaccine in their childhood.

The present study is a long-term follow-up to a primary study that has confirmed the non-inferiority of a two-dose schedule of the *Adult *formulation of this hepatitis B vaccine versus a three-dose schedule of the *Paediatric *formulation, when comparing the anti-HBs seroprotection rates and anti-HBs antibody GMCs at Month 7 [4]. This follow-up study evaluated the five year persistence of antibodies against hepatitis B surface (anti-HBs) antigens in adolescents who received the two-dose regimen of this hepatitis vaccine compared to those who received the three-dose regimen, and the ability of these subjects to mount an anamnestic response to a challenge dose of hepatitis B vaccine given five years after completion of primary immunisation.

## Methods

### Study design and subjects

In 2001, healthy adolescents aged between 11 and 15 years were enrolled into a single-blind, randomised, multi-country study conducted in Belgium, Australia and Ukraine. The subjects (randomisation blocking scheme 2:1) received either two doses of *Engerix*-B™ *Adult *formulation (20 μg of recombinant hepatitis B surface antigen [HBsAg], thiomersal-free formulation) following a 0, 6 months schedule [Group HBV_2D] or three doses of *Engerix*-B™ *Paediatric *formulation (10 μg of recombinant HBsAg, preservative-free formulation) following a 0, 1, 6 months schedule [Group HBV_3D]. Group HBV_2D additionally received an injection of physiological saline as placebo at second vaccination time point (Month 1) to maintain the blinding. The vaccines were administered as deep intramuscular injections (needle-length: 25 mm; gauge: 23) in the deltoid region of the arm [4].

These subjects were then followed up for the next five years (until Year 5 time point) with pre-defined annual visits to evaluate the persistence of anti-HBs antibodies induced by the two schedules of the hepatitis B vaccine [NCT00343915]. Subjects who had completed the primary vaccination course of hepatitis B vaccines and met the eligibility criteria for the challenge phase were administered a challenge dose of hepatitis B vaccine (10 μg of recombinant HBsAg, preservative-free formulation) 72-78 months later, were evaluated one month later for immune memory to the HBs antigen [NCT00524576]. Subjects were excluded from the challenge phase if they had used any investigational product within 30 days preceding the hepatitis B challenge dose or received/planned to receive any vaccines unforeseen by the protocol within 30 days preceding or post-hepatitis B challenge dose or received an additional dose of hepatitis B vaccine between the primary and challenge phases; subjects were also excluded if they had confirmed of suspected diagnosis of immunosuppressive or immunodeficient condition. Pregnant or lactating female subjects were also excluded.

The study was conducted respecting the Good Clinical Practice (GCP) guidelines and Declaration of Helsinki. Two of the four centres were eliminated from the evaluation of anamnestic response to the challenge dose; the investigator from the study centre was not confident that their team would be able to recruit a sufficient number of subjects and therefore did not participate in this phase of the study; while the subjects at another study centre were excluded from the primary analysis due to GCP non-compliance.

The study protocol was approved by the independent ethics committees of the Children's Hospital at Westmead, Antwerp University Hospital, UZA Wilrijkstraat, HopitaloFacultaire de I'UCL and State Entreprise Centre of Immunobiological Preparation; written informed consent was obtained from the parents/guardians of adolescent subjects and from subjects who were above 18 years of age before conducting any study-related procedure.

### Serological assessment

Anti-HBs antibody concentrations were measured in the serum samples collected at the first two follow-up visits (at Years 2 and 3 time points) using a commercial enzyme immunoassay (AUSAB EIA/Abbott; cut-off: ≥3.3 mIU/mL). For the subsequent visits (at Years 4 and 5 time points) and the challenge visits, an in-house quantitative enzyme-linked immunosorbent assay (cut-off: ≥3.3 mIU/mL) was used; this assay was equivalent to the previously available commercial assay and was fully validated by calibrating against the first International Reference Preparation for anti-hepatitis B immunoglobulin [14]. All evaluations were done at the Central laboratory, GSK Biologicals, Belgium, except for the last visit (at Year 5 and challenge visits), for which the evaluations were conducted at the CEVAC laboratory, Ghent University and Hospital, Ghent, Belgium.

The percentage of subjects with anti-HBs antibody concentrations ≥10 mIU/mL, GMCs, GMC evolution at all follow-up time points was tabulated with 95% CI.

### Assessment of safety

Safety assessment of the challenge dose included prospective reporting of solicited local and general adverse events and serious adverse events (SAEs). The adverse events were graded on a three-point scale, with those adverse events that hampered normal daily activities being graded as Grade 3 symptoms.

### Statistical analyses

No separate sample size calculations were conducted for the long-term follow-up phase of the study. All subjects who participated in the primary study were invited to participate in the subsequent long-term follow-up time points, subject to them meeting the inclusion and exclusion criteria.

The primary analyses of immunogenicity at the long-term follow-up time points (all four centres) were performed on the long-term ATP cohort for immunogenicity, while for the challenge phase, the analyses were performed on the ATP cohort for immunogenicity. Assessments of safety for the challenge phase were performed on the total vaccinated cohort (TVC).

The long-term ATP cohort for analyses of immunogenicity included those subjects who were part of the ATP cohort for immunogenicity analyses in the primary study, with available results from the long-term follow-up time points and without any protocol violations. The ATP immunogenicity cohort for the challenge phase included those subjects who were protocol-compliant and for whom post-challenge dose data was available.

An anamnestic response to the challenge dose was defined taking into consideration the pre-challenge dose serostatus of subjects; for seropositive subjects, an anamnestic response was defined as a four-fold increase in anti-HBs antibody concentration, and for seronegative subjects, as an anti-HBs antibody concentrations ≥10 mIU/mL post-challenge.

GMCs were calculated by taking the anti-log of the mean of log-transformed anti-HBs antibody concentration values.

All statistical analyses were performed using Statistical Analysis System (SAS) version 9.1 and StatXact-7 on SAS.

## Results

### Study population

**Figure 1:**
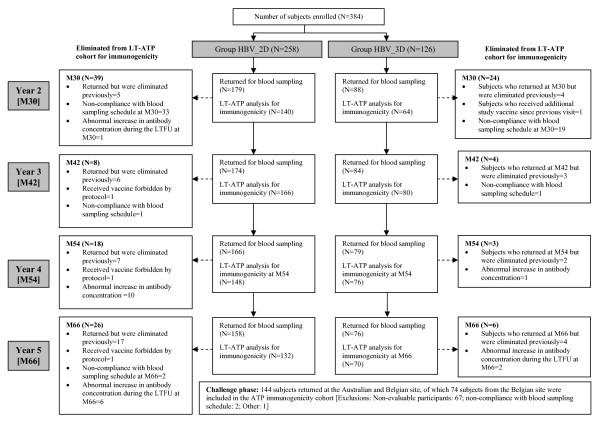
**CONSORT diagram**.

The five years follow-up period concluded in January 2008 and a total of 234 subjects were available for this follow-up visit. The challenge phase which concluded in May 2008 included a total of 144 subjects. The number of subjects who participated at each time point during the long-term follow-up and the challenge phase is presented along with the reasons for non-participation of other subjects in Figure 1.

The demographic characteristics in both groups were similar at the time of recruitment for the primary vaccination phase, at the Year 5 time point as well as at the time of the challenge dose. In addition, there was no difference in demographic characteristics across the centres in the three countries, at study start. Five years after the primary immunisation, the overall mean age was 18.5 ± 1.44 years (range: 16-21 years) and 51.7% of subjects were females; all subjects except two (99.1%) were of Caucasian origin. At the time of the challenge dose, mean age was 19.5 ± 1.30 years (range: 17-22 years); 50.7% of subjects were females and all subjects except three (97.9%) were of Caucasian origin. The exclusion of study centres in the challenge phase did not have any impact on the demographic profile of the population, as compared to the primary phase where 50.3% of subjects were females and all except 11 subjects (97.1%) were of Caucasian origin.

### Immunogenicity

#### Antibody persistence

For the long-term follow-up period, the primary analyses of immunogenicity (long-term ATP cohort for immunogenicity) were conducted on subjects from all centres, while for the challenge phase, the primary analyses of immunogenicity and safety were performed on subjects from two out of the four centres (ATP cohort for immunogenicity).

**Figure 2:**
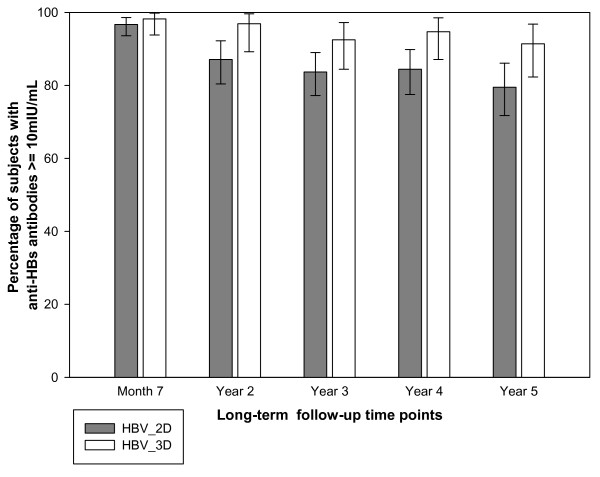
**Percentage of subjects with anti-HBs concentration ≥10 mIU/mL at all long-term follow-up time points (Long-term ATP cohort for immunogenicity)**.

**Figure 3:**
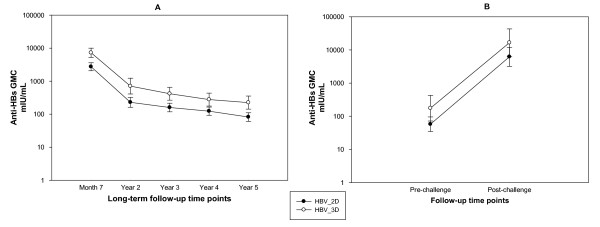
**Evolution of anti-HBs antibody geometric mean concentrations (GMCs) during the five years follow-up period (Long-term ATP cohort for immunogenicity) and during the challenge phase (ATP cohort for immunogenicity)**.

Five years after the primary immunisation, 79.5% (95% confidence interval [CI]: 71.7 - 86.1) of subjects in the HBV_2D group and 91.4% (95% CI: 82.3 - 96.8) of subjects in the HBV_3D group had anti-HBs antibody concentrations ≥10 mIU/mL (overlapping 95% confidence intervals) (Figure 2). The anti-HBs antibody GMCs (calculated on seropositive subjects) at all follow-up time points appeared to be higher in subjects in the HBV_3D group compared to those in the HBV_2D group (non-overlapping 95% confidence intervals). The rate of decrease of antibody concentrations was similar in both groups, as shown by the parallel slopes of the GMC kinetic curve (Figure 3A).

#### Immune memory

One month after the challenge dose, all subjects (100%) in both groups had anti-HBs antibody concentrations ≥10 mIU/mL and a similar proportion of subjects in both groups had anti-HBs antibody concentration ≥100 mIU/mL (HBV_2D: 94.3% [95% CI: 84.3 - 98.8]; HBV_3D: 95.2% [95% CI: 76.2 - 99.9]).

All subjects (100%) in both groups mounted an anamnestic response to the challenge dose of hepatitis B vaccine irrespective of their pre-challenge dose serostatus. All subjects who had diminishing levels of anti-HBs antibodies before the challenge dose (<3.3 mIU/mL or 3.3-10 mIU/mL [pooled: seven and six subjects, respectively]) showed anti-HBs antibody concentrations ≥10 mIU/mL after the challenge dose. In comparison, subjects who had robust pre-challenge anti-HBs antibodies concentrations (≥10 mIU/mL and ≥100 mIU/mL [pooled: 35 and 26 subjects, respectively]) continued to have similarly high anti-HBs antibody levels post-challenge dose (Table 1). In both groups, there was a large increase in the GMCs one month post-challenge dose (HBV_2D: 6214.1 mIU/mL [108-fold increase, approximately]; HBV_3D: 16564.3 mIU/mL [95-fold increase, approximately]) (Figure 3B).

**Table 1:** Anti-HBs immune response in subjects according to their pre-challenge dose serostatus (ATP cohort for immunogenicity)

Group	Pre-challenge status (mIU/mL)	N	≥10 mIU/mL	≥100 mIU/mL	GMC
**% or Value (95% CI)**					

**HBV_2D**	<3.3	6	100 (54.1 - 100)	66.7 (22.3 - 95.7)	277.5 (24.7 - 3117.3)
	3.3 to <10	6	100 (54.1 - 100)	83.3 (35.9 - 99.6)	821.7 (142.8 - 4729.5)
	10 to 100	26	100 (86.8 - 100)	100 (86.8 - 100)	6117.3 (2875.1 - 13015.7)
	≥100	15	100 (78.2 - 100)	100 (78.2 - 100)	49745.4 (24663.9 - 100332.9)

**HBV_3D**	<3.3	1	100 (2.5 - 100)	0.0 (0.0 - 97.5)	37.4 (-)
	3.3 to <10	0	-	-	-
	10 to 100	9	100 (66.4 - 100)	100 (66.4 - 100)	6048.5 (2414.6 - 15150.9)
	≥100	11	100 (71.5 - 100)	100 (71.5 - 100)	65723.7 (34920.1 - 123699.6)

**Pooled**	<3.3	7	100 (59.0 - 100)	57.1 (18.4 - 90.1)	208.4 (26.3 - 1648.7)
	3.3 to <10	6	100 (54.1 - 100)	83.3 (35.9 - 99.6)	821.7 (142.8 - 4729.5)
	10 to 100	35	100 (90.0 - 100)	100 (90.0 - 100)	6099.5 (3396.3 - 10954.1)
	≥100	26	100 (86.8 - 100)	100 (86.8 - 100)	55967.0 (35483.1 - 88276.2)

N = number of subjects with available results; 95% CI = exact 95% confidence interval

GMC = geometric mean concentrations

As a post-hoc analyses, to ensure that the subjects participating in the challenge phase of the study were representative of the initial population that was enrolled in the primary study, the post-primary immune response in subjects included in the ATP cohort for immunogenicity in the challenge phase was compared to that in excluded subjects and found to be comparable irrespective of whether they received the two-dose or three-dose schedules (overall percentage of subjects with anti-HBs antibody concentration ≥10 mIU/mL participants: 97.1% [95% CI: 90.1 - 99.7]; non-participants: 97.2% [95% CI: 94.5 - 98.8]; anti-HBs antibody GMCs: 3037.2 mIU/mL and 3939.8 mIU/mL, respectively).

#### Safety and reactogenicity

The challenge dose was generally well-tolerated. Pain at the site of injection and fatigue were the most frequently reported solicited local and general symptoms, respectively (33.8% of subjects reported each). None of the subjects reported local symptoms of Grade 3 intensity, and one subject (1.3%) reported a general symptom, fatigue, of Grade 3 intensity. Five subjects reported unsolicited symptoms that were considered by the investigator to be vaccine-related. These symptoms were vision impairment (diagnosed to be transient and due to fatigue), injection site paraesthesia, myalgia, allergic dermatitis and rash (one subject each). One subject reported Grade 3 unsolicited symptoms, myalgia that was considered by the investigator to be vaccine-related. All these adverse events had resolved by the end of the study. No SAEs were reported following the challenge dose.

## Discussion

The primary study had established that the immunogenicity of a two-dose regimen of the *Adult *formulation of hepatitis B vaccine was non-inferior to that of a three-dose regimen of the *Paediatric *formulation. Overall, both regimens had comparable safety profiles [4]. In order to address concerns regarding the possibility of waning immune memory against the hepatitis B antigen over time, it was essential to evaluate the duration of persistent immunity following the two-dose hepatitis B primary immunisation.

Studies assessing the immunogenicity of two- and three-dose primary vaccination against hepatitis B in adolescents have reported seroprotection rates between 93.4% and 99.5% and anti-HBs antibody GMCs up to 4155 mIU/mL [12,13,15]. Data on the long-term persistence of anti-HBs antibodies in adolescents following a three-dose schedule as obtained from two published long-term follow-up studies had established that 94.1% of adolescents retained anti-HBs antibody concentrations ≥10 mIU/mL for at least five years, while 91.2% of adolescents retained anti-HBs antibody concentrations ≥10 mIU/mL up to 10 years after completion of the primary vaccination schedule [13,16]. The findings from the present study are in line with these previous long-term follow-up studies. In this study, the anti-HBs antibodies persisted for at least five years after primary vaccination, irrespective of whether the subjects received the two- or three-dose schedules. At the end of the long-term follow-up period, 79.5% and 91.4% subjects in the HBV_2D and HBV_3D groups, respectively showed anti-HBs antibody concentrations ≥10 mIU/mL.

The anti-HBs antibody GMC evolution observed in the present study is similar to observations from previous studies [2,11], where the GMCs declined rapidly in the first year after primary vaccination, followed by a more gradual decrease over the subsequent years. The anti-HBs antibody GMC observed in the HBV_3D group was comparatively higher than that observed in the HBV_2D group, at all follow-up time points. However, the evolution of anti-HBs antibody GMC observed throughout the follow-up period was similar in both groups. Of note, the fold increase in anti-HBs antibody GMC following the challenge dose was slightly higher in the HBV_2D group than in the HBV_3D group (108-fold and 95-fold, respectively). Thus, it is evident that the subjects in both groups, irrespective of their anti-HBs antibody levels prior to the challenge dose had sufficient immune memory to mount an effective anamnestic response to the challenge dose administered five years after completion of the primary vaccination course.

These results are consistent with previous long-term studies with two- and three-dose schedules of the hepatitis B study vaccine which have reported that five to ten years after primary vaccination, between 81.0% and 99% of children and adults had anti-HBs antibody concentrations ≥10 mIU/mL [17-19]. In addition, the fact that all subjects in the present study could mount an anamnestic response to the challenge dose indicates strong immune memory against the hepatitis B vaccine antigen.

A potential weakness of this study is that the sample size calculation was based on the objective of the primary study (to compare the immune response induced by the Adult and Paediatric formulations of the hepatitis B study vaccine following a two-dose or three-dose primary vaccination course) and hence did not account for the attrition of subjects over a period of five years. However, the population at the start of the study and the population followed up were comparable in terms of anti-HBs response to primary vaccination and demographic characteristics, as evident from the post-hoc analysis. Therefore there was no bias in the selection of the final study cohort.

The observations from this study are in line with previous reports that the decrease in anti-HBs antibody concentrations to even undetectable levels does not necessarily indicate loss of protection in the long-term and that immunological memory can outlast the loss of antibodies [1,20]. The data from two separate studies in infants and adolescents that evaluated persistence of anti-HBs antibodies five and ten years after primary vaccination have further established these observations [13,21]. The fact that the vaccinees with undetectable levels of anti-HBs antibodies or waning antibody levels responded with an anamnestic response to the challenge dose indicate that there is currently no evidence that booster dose of hepatitis B vaccine is required after a successful primary vaccination [13,21].

A two-dose schedule of GSK Biologicals' *Engerix*-B™ *Adult *(anti-HBsAg content: 20 μg), which has a good safety and immunogenicity profile and is generally well-tolerated, is therefore a suitable alternative to the standard three-dose schedule of the *Paediatric *formulation (for adolescents aged 11+) and may facilitate higher immunisation completion rates [21-24] with the reduction in the required number of injections and clinical visits. In addition, a catch-up regimen of a two-dose schedule in older adolescents susceptible to the disease and in whom compliance with a three-dose *Paediatric *dosing schedule is in doubt, may also be used in order to improve population-based immunity [21].

## Conclusions

The two-dose schedule of the *Adult *formulation of hepatitis B vaccine when administered to adolescents induced persistence of detectable anti-HBs antibodies for at least five years after completion of the primary vaccination schedule. The strong anamnestic response following the challenge dose regardless of the priming schedules provides the evidence of strong immunological memory for at least five years following vaccination.

## List of Abbreviations

HCC: Hepatocellular carcinoma; WHO: World Health Organization; GSK: GlaxoSmithKline; anti-HBs: Antibodies against hepatitis B surface antigens; CEVAC: Centre for Evaluation of Vaccines; SAE: Serious adverse events; ATP: According to protocol; TVC: Total vaccinated cohort; SAS: Statistical Analysis System.

## Competing interests

PVD acts as chief and principal investigator for clinical trials conducted on behalf of the University of Antwerp, for which the University obtains research grants from vaccine manufacturers; speaker's fees for presentations on vaccines are paid directly to an educational fund held by the University of Antwerp. AM, IM and AK declare to have no conflict of interest. RB declares that occasionally, organisations such as CSL, Roche, Sanofi Pasteur, GSK and Wyeth Lederle have provided funding to RB to attend and present at scientific meetings; RB also has received financial support from various organisations to conduct research - any funding received is directed to a research account at The Children's Hospital at Westmead and is not personally accepted by RB. SK, AB, SN, ML and JJ are currently employed at GSK Biologicals; AB, SN, ML and JJ also have stock ownership at GSK Biologicals.

## Authors' contributions

PVD participated in the design of the study, acquisition of the data and interpretation of the data; AM, IM, AK, RB, SK, AB, SN, ML and JJ have been involved in the acquisition of data and interpretation of the data. SK performed the statistical analysis. All authors were involved in critical review and have commented on the draft manuscripts; and have read and approved the final manuscript.

## Pre-publication history

The pre-publication history for this paper can be accessed here:

http://www.biomedcentral.com/1471-2334/10/357/prepub

## Trademark Statement

*Engerix*-B is a trademark of GlaxoSmithKline group of companies.
